# What happens when chitin becomes chitosan? A single-molecule study†

**DOI:** 10.1039/d2ra07303j

**Published:** 2023-01-16

**Authors:** Lu Qian, Kai Zhang, Xin Guo, Miao Yu

**Affiliations:** a School of Mechanical Engineering, Sichuan University Chengdu 610065 China miaoyu@scu.edu.cn; b School of Materials Science and Engineering, South China University of Technology Guangzhou 510641 China

## Abstract

Chitin and chitosan are important support structures for many organisms and are important renewable macromolecular biomass resources. Structurally, with the removal of acetyl group, the solubility of chitosan is improved. However, the specific mechanism of solubility enhancement from chitin to chitosan is still unclear. In this study, the atomic force microscopy (AFM)-based single molecule force spectroscopy (SMFS) was used to obtain the single-chain mechanical behavior of chitin and chitosan. The results show that the hydrogen (H)-bonds' state, which can be influenced by the solvent, determines the degree of binding water (solubility) of polysaccharides, and that the binding water energy of a single chitosan chain is 6 times higher than that of chitin in water. Thus, H-bonding is the key to solubility enhancement and can be used to modulate the solubility properties of chitosan. It is expected that our studies can help to understand the structural and functional properties of chitin and chitosan at the single molecule level.

## Abstract 2

Single molecule studies showed that the hydrogen (H)-bonds'state, which can be influenced by the solvent, determines the degree of binding water of polysaccharides.

## Graphical abstract

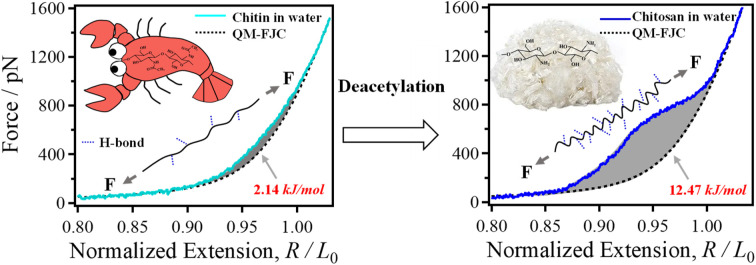

## 1 Introduction

Renewable biomass is one of the important solutions to the energy problem under the consideration of “sustainable development” and “environmental protection”. Among the renewable biomasses, natural polymers occupy an important position, of which chitin and chitosan, as typical biological resources, are of great value for development and utilization. Chitin, as one of most important biopolymers, is a chain-like polymer consisting of *N*-acetyl glucosamine.^1,2^ As an ancient and important structural polysaccharide, chitin is an important component of life.^3–5^ Chitosan is an important product of chitin after deacetylation,^6^ which show distinctive physicochemical (such as the chemical activity of chitosan is higher than chitin) and biological properties due to the presence of amine groups.^1,7^ As the only natural cationic polyelectrolyte polysaccharide, chitosan shows wide application in the food industry, biomedical industry, cosmetics industry, *etc.*^8–10^

The structure and function of polymers are closely related to the polymorphs, and previous studies have shown that chitin exists in abundant polymorphs. According to the different sources in natural, chitin can be divided into three polymorphs: α-, β-, and γ-chitin.^11,12^ α-chitin is the most abundant and the most stable structure among the three polymorphs. Chitin is always as structural materials in insect cuticles, shells of crabs, lobsters and shrimp, and in fungal/yeast cell walls.^13^ Apart obtained from nature, α-chitin can also be obtained by recrystallization from the chitin solution or biosynthesis.^14,15^ The amount of β-chitin is less than that of α-chitin, and the structure stability of β-chitin is weak than that of α-chitin.^16^ Up to now, the β-chitin can only be obtained from nature (such as squid pens).^2^ γ-chitin, complex of α and β polymorphs, can be obtained from the cocoons of beetles and the stomachs of squid.^17–19^ α-, β-, and γ-chitin, correspond to anti-parallel, parallel, and alternated arrangements of polymer chains, respectively.^20,21^ In crystals, the chitin chains adopt the sheets structure, which was tightly fixed by the strong intra-sheet interactions such as C–O⋯NH hydrogen bonds (H-bond). The inter-sheet H-bonds between adjacent chains are also existed in α-chitin.^22^ However, these inter-sheet H-bonds cannot be found in β-chitin.^2,23^ Chitosan present the semi-crystalline structure in solid state. Single chitosan crystals can be obtained from the fully deacetylated chitin. In this structure, two antiparallel chains can be observed in the unit cell.^24^ Thus, as important biomasses with potential applications, the structural and functional relationships of these two polysaccharides have become the target of extensive research, as well as an important approach to promote the application of the two polysaccharides.

Chitin is highly hydrophobic and with strong intra- and intermolecular H-bonds, which result in insoluble properties of this polymer in common solvents at room temperature, for example, water, acidic and alkali aqueous solution.^25^ It is soluble in highly concentrated inorganic acids, *N*,*N*-dimethylacetamide DMA and LiCl mixture, lithium thiocyanate, *etc.* However, most of these solvents are environmentally unfriendly, which hindered the development of the chitin industry.^26,27^ Chitosan shows a better solubility than chitin, which can be dissolved in acidic aqueous solution (whereby the chitosan is converted to a polyelectrolyte).^2,5^ Due to the relatively good solubility in aqueous solution, chitosan was widely used as hydrogels and films, which physico-chemical properties can be effectively affected by temperature, pH and degree of acetylation *etc.*^28–31^ Meanwhile, chitosan show a good metal ions capture ability, which present a great advantage for chitosan in textile wastewater treatment. Chitin and chitosan show largely differences in the crystal structure, solubility, degradability, biological functions, *etc.* However, from chitin to chitosan, researchers have mostly focused on the applied properties of the two polysaccharides, and few studies have paid attention to the differences of these polysaccharides at the single molecule level, which is the key to determine their macroscopic properties.

Atomic force microscopy (AFM)-based single molecule force spectroscopy (SMFS) is a powerful tool to investigate the properties of biomacromolecules (such as DNA, proteins and cellulose) from the single molecule level.^32–46^ AFM has been used to study the successful in this study, AFM-based SMFS was used to study the single-chain elasticity of chitin and chitosan in different conditions to investigate the similarities and differences from chitin to chitosan. The force measurements results indicate that these two polysaccharides present the same strength of intrachain H-bonds in the same solvents (nonane or DMSO), which results in these two polysaccharides present the same single-chain natural and backbone elasticity in the same solvents. However, there are remarkable differences for the nanomechanics and the properties of H-bonds of chitin and chitosan in water. This study cast a new insight into the roles of H-bonds and water in the physical and biological properties of these important polysaccharides at the single-molecule level.

## 2 Experimental section

### 2.1 Materials and chemicals

The target polysaccharides of in this research are chitin (from shrimp shells, CAS: 1398-61-4, 83% degree of acetylation, Aladdin, China) and chitosan (CAS: 9012-74-6, ≥ 95% degree of deacetylation, Aladdin, China). The molecular weight of these two polysaccharides is 1400–4000 kDa. The ionic liquid 1-allyl-3-methylimidazolium chloride (AMIMCl) (CAS: 65039-10-3, Aladdin, China), nonane and DMSO were purchased from Sigma-Aldrich (China). The chemicals used in this study are analytically pure.

### 2.2 Sample preparation

The concentration of polysaccharides in this study is 1 mg L^−1^. The chitin and chitosan are dissolved in AMIMCl (70 °C) and water (60 °C) respectively. Glass slides are used as substrate in this study. Before use, the substrates are treated with a mixture solution of 98% H_2_SO_4_ and 35% H_2_O_2_ (7 : 3, v/v) with a temperature of 90 °C for 40 min, and then the glass slides are washed with water and dried in air. The glass slides are immersed in chitin or chitosan solution for 20 min, and then the substrates are washed with water to remove the poorly adsorbed molecules. Finality, the prepared substrate was blown dry and used immediately for the SMFS experiments.

### 2.3 Force measurements

The AFM used in this study is MFP-3D (Asylum Research) (Santa Barbara, CA, USA). The AFM tip is purchased from Bruker (MSCT-D) with V-shaped (Si_3_N_4_). Thermal excitation method was used to calibration the spring constant (range from 30 to 40 pN nm^−1^) of the AFM tip. The temperature was maintained at 23 °C during the experiment and the loading rate was 2 μm s^−1^. Multiple replicate experiments (at least 3 substrates of each condition) were used to ensure the credibility of the experimental data. Instrumentation details of SMFS can be found elsewhere.^47,48^

## 3 Results and discussion

### 3.1 The similar single-chain elasticity of chitin and chitosan in organic solvents

#### 3.1.1 The single-chain natural elasticity of chitin

**Fig. 1:**
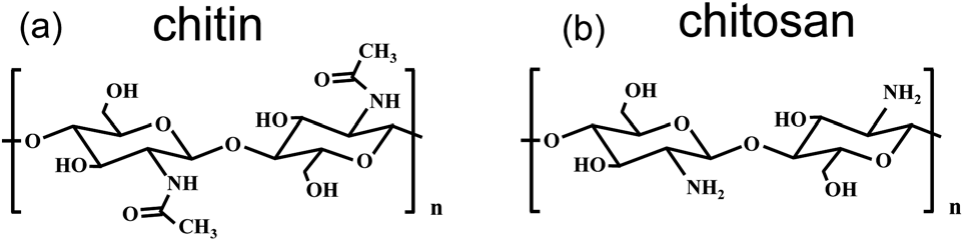
The molecular structure of chitin (a) and chitosan (b).

**Fig. 2:**
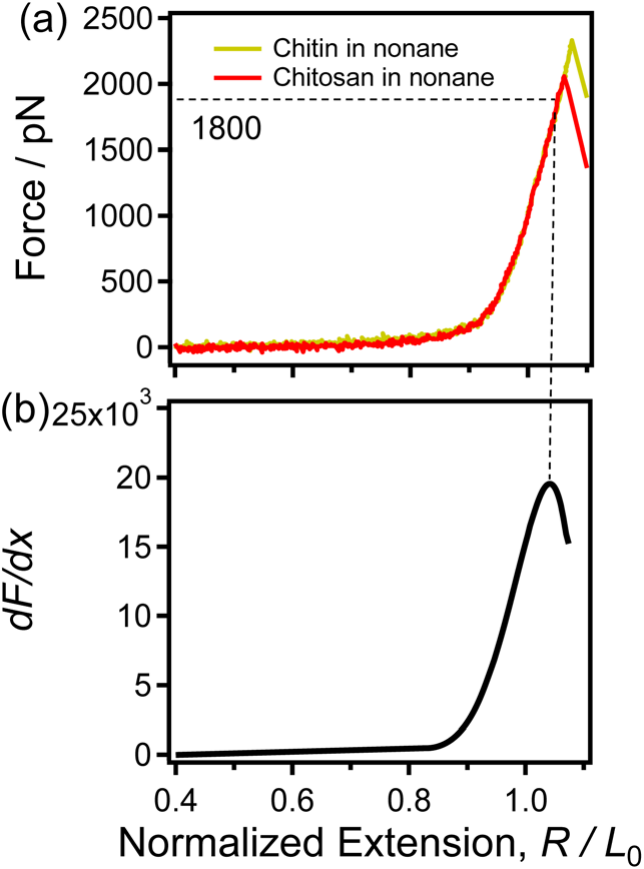
(a) The normalized single-chain F-E curves of chitin (yellow) and chitosan (red) obtained in nonane respectively. (b) The d*F*/d*x* of the F-E curve that shown in (a).

As shown in Fig. 1, chitin shows a molecular structure similar to that of chitosan. Thus, it can be speculated that the two polysaccharides should also have many similarities in single-chain properties. Previous studies reported that the nonpolar organic solvent is a good environment to investigate the single-chain elasticity of polymers under an undisturbed condition due to the interactions between a polymer and nonpolar organic solvent molecules is van der Waals forces, which can be ignored in force measurements.^49^ The single-chain elasticity of polymer obtained in nonpolar organic solvent can be described as the “single-chain natural elasticity”. Thus, a typical nonpolar organic solvent (nonane) used as the experimental condition to investigate the natural elasticity of the two polysaccharides. Fig. 2a and Fig. S1† show the force-extension (F-E) curves of chitin and chitosan obtained in nonane. See Fig. 2a, the superposition of the normalized F-E curves indicating that chitin and chitosan present the same natural elasticity of in this condition.^50^

Moreover, in recent studies, the derivatives (d*F*/d*x*) of the normalized F-E curves can provide more information (such as the force characterizing the transition of the polymer chain) for studying the single-chain elasticity of polymer.^51,52^ As shown in Fig. 2b, the d*F*/d*x* of the F-E curves in Fig. 2a increases firstly and then decreases. Previous studies have demonstrated that the turning point corresponds to the structural transition (conformation transition or broken of intrachain H-bonds) of the polymer upon stretching.^51,53^ It has been reported that the conformation of sugar rings does not change for the β-(1–4) glycosidic bonds linked polysaccharides (*i.e.*, cellulose) upon stretching.^54,55^ In addition, chitin and chitosan share the same linkage between adjacent sugar rings, *i.e.*, β-(1–4)-linked d-glucosamine. Thus, it can be speculated that the turning point in Fig. 2b cannot be caused by the conformation transition. In the nonpolar solvent nonane, chitin/chitosan exhibit natural elasticity. Chitin/chitosan can form extensive intrachain H-bonds due to large number of H-bonds donor/acceptor (with abundant hydroxyl groups, amido, acetyl and oxygen atoms) and the weak disturbance from solvent. Therefore, it can be speculated that the turning point in Fig. 2b caused by the fracture of the intrachain H-bonds chitin/chitosan upon stretching in nonane. In Fig. 2, the force is about 1800 pN correspond to the turning point, indicating the break force of intrachain H-bonds is 1800 pN. This rupture force is remarkable larger than that of the strength of the traditional H-bonds involved in polymer. The abundant intrachain H-bonds of the cellulose and poly(vinyl alcohol) systems are reported to synergistically enhance the single-chain mechanics of these polymers, which makes the intrachain H-bonds of these polymers significantly stronger than the traditional H-bonds (several to dozens of pN).^51,56^ Thus, it can be speculated that the large rupture force (1800 pN) of chitin/chitosan in nonane is attribute to the synergistic enhancement effect of intrachain H-bonds.

#### 3.1.2 The single-chain backbone elasticity

DMSO is an extremely important nonpolar polar solvent, and is known as a “universal solvent”. It is widely used as a solvent and reaction reagent with high selective extraction ability and denaturation of proteins. Previous single-molecule experiments have shown that DMSO can eliminate almost all the intrachain H-bonds of polysaccharides (*e.g.* natural cellulose and amylose).^57^ Therefore, DMSO was used as an environmental factor eliminator to investigate the single-chain mechanics of chitin and chitosan related only to their backbones, which is called “single-chain backbone elasticity”.

**Fig. 3:**
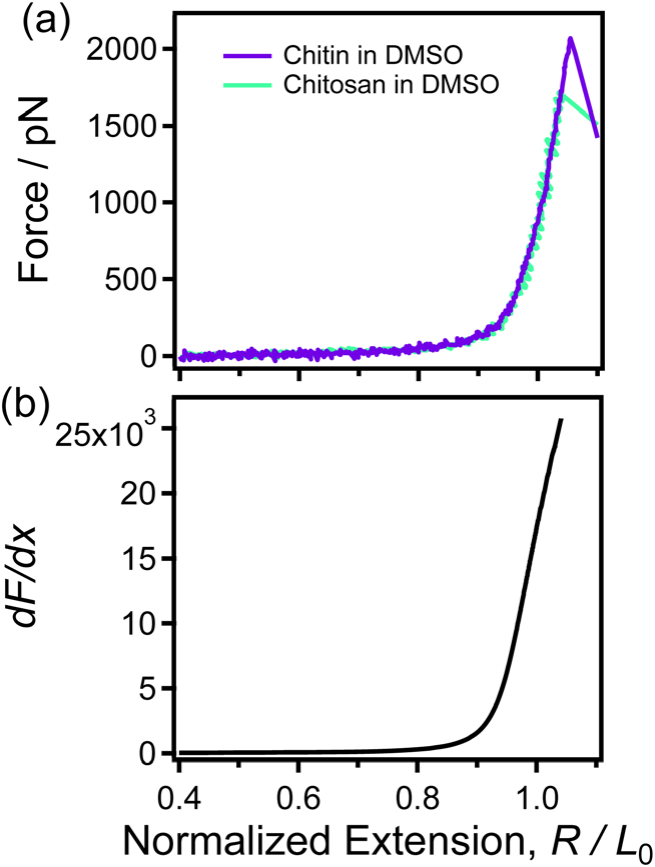
(a) The normalized single-chain F-E curves of chitin (purple) and chitosan (green) obtained in DMSO respectively. (b) The d*F*/d*x* of the F-E curve that shown in (a).

As shown in Fig. S2,† the normalized F-E curves of chitin/chitosan superposed well, indicating that the single-chain elasticities of chitin and chitosan in DMSO were observed respectively. Furthermore, Fig. 3a shows that the F-E curves of chitin and chitosan obtained in DMSO superposed in whole force region, indicate that the two polysaccharides exhibit the same backbone elasticity in DMSO. By studying the inherent elasticity of polymers with carbon–carbon (C–C) backbone, Cui *et al.* reported that side chains show little effect on the single-chain inherent elasticity of polymers,^58,59^ and polymers with the same backbone have the same inherent elasticity (*i.e.* the backbone elasticity in this paper). This result is consistent with our results that chitin and chitosan have the same backbone elasticity with the absence of strong intrachain interactions (intrachain H-bonds).

Fig. 3b shows that the d*F*/d*x* of the F-E curve obtained in DMSO is increases monotonically in the whole range, which clearly different from the case in nonane (Fig. 2b). This means that there is no structural change when single chitin/chitosan chain is stretched in DMSO. In other words, chitin and chitosan cannot form intrachain H-bonds in DMSO. Therefore, it can be concluded that the groups of sugar rings cannot affect the single-chain backbone elasticity of polysaccharide with same backbone. These results indicate that chitin show the same statue of H-bonds and single-chain mechanics with chitosan in same solvent (nonane or DMSO).

#### 3.1.3 Effect of H-bonds on the single-chain elasticity of chitin/chitosan in organic solvents

Theoretical calculation can provide a deeply understanding for investigating the single-chain stretching process of polymer. Previous studies shown that the stretching process amylose and cellulose can be well described by several theoretical models.^57,60,61^ In this work, the quantum mechanical modified freely joint-chain (QM-FJC) model was used to study the single chain behavior of chitin/chitosan upon elastic elongation. In this model, the relationship between the extension (*R*) and force (*F*) can be written as follows:

1 *R*/*L*_0_ = (*L*[*F*]/*L*_0_){coth[(*FL*_k_)/(*k*_B_*T*)] − (*k*_B_*T*)/(*FL*_k_)}

Where *R*/*L*_0_ is the normalized extension of a polymer, *L*[*F*] and *L*_0_ are the contour lengths when force equal to *F* and zero respectively. *L*_k_ is the Kuhn length, *k*_B_ is the Boltzmann constant, and *T* is the temperature. The *L*_k_ is 0.514 nm, which is has been used to calculate the single-chain elasticity of polysaccharides that consist of β-(1–4)-d polysaccharides.^60,62^

**Fig. 4:**
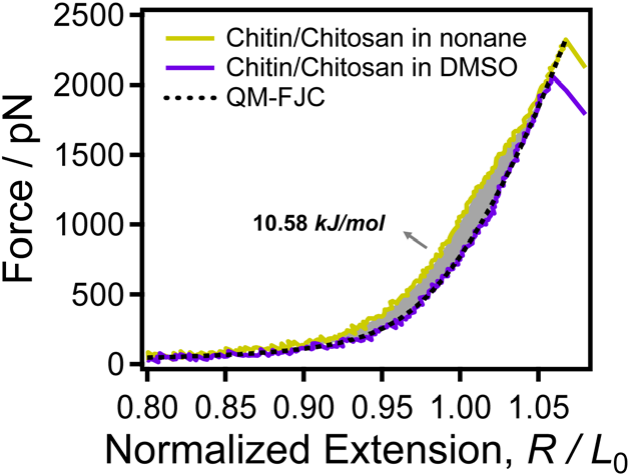
The normalized F-E curves of chitin/chitosan obtained in nonane (yellow) and DMSO (purple) respectively. The black dotted line is the QM-FJC model fitting curve.

See Fig. 4, the theoretical curve (black dotted line) is superposed well with the experimental curve of chitin/chitosan in (purple solid line) in the whole force region. Previous studies indicate that the QM modified models only can be used to describe the backbone elasticity of polymer, the effects of solvents and interaction between side chains cannot be considered.^60^ Thus, the superposition of the theoretical model and experimental curves implies that the backbone elasticity of chitin/chitosan can be obtained in DMSO. In other words, DMSO can eliminate almost all the intrachain H-bonds of chitin/chitosan.

In nonane, the experimental curve of chitin/chitosan (yellow solid line) superposed with the QM-FJC curve both in low force region (*F* < 50 pN) and high force region (*F* > 2000 pN). While, the curve obtained in nonane is higher than the QM-FJC theoretical curve in the middle force region. Previous studies had showed that single chitin chain tends to form extensive intra-chain H-bonds in nonane.^63^ Therefore, it can be confirmed that the difference (the grey area) between the QM-FJC curve and the experimental curve of chitin/chitosan in nonane is closely related to intrachain H-bonds. The area formed by the F-E curve and the coordinate axis is the energy consumed in the stretching process.^64^ That is to say, the larger the area the higher the energy required for the stretching process, the more the chain is constrained. The calculation result shows that the energy difference between the natural elasticity and the backbone elasticity of chitin/chitosan is 10.58 kJ mol^−1^, *i.e.* the energy generated by breaking the intrachain H-bonds of chitosan chain is 10.58 kJ mol^−1^. These results demonstrate that the solvent deeply involved in the single-chain elasticity of chitin/chitosan by regulating H-bonds.

These force measurements indicate that chitin and chitosan show the same strength of H-bonds and the same single-molecule mechanics in the same solvent (nonane or DMSO) even if the groups in C-2 position are different (amido or acetyl) between them.

### 3.2 The difference single-chain elasticity of chitin and chitosan in water

**Fig. 5:**
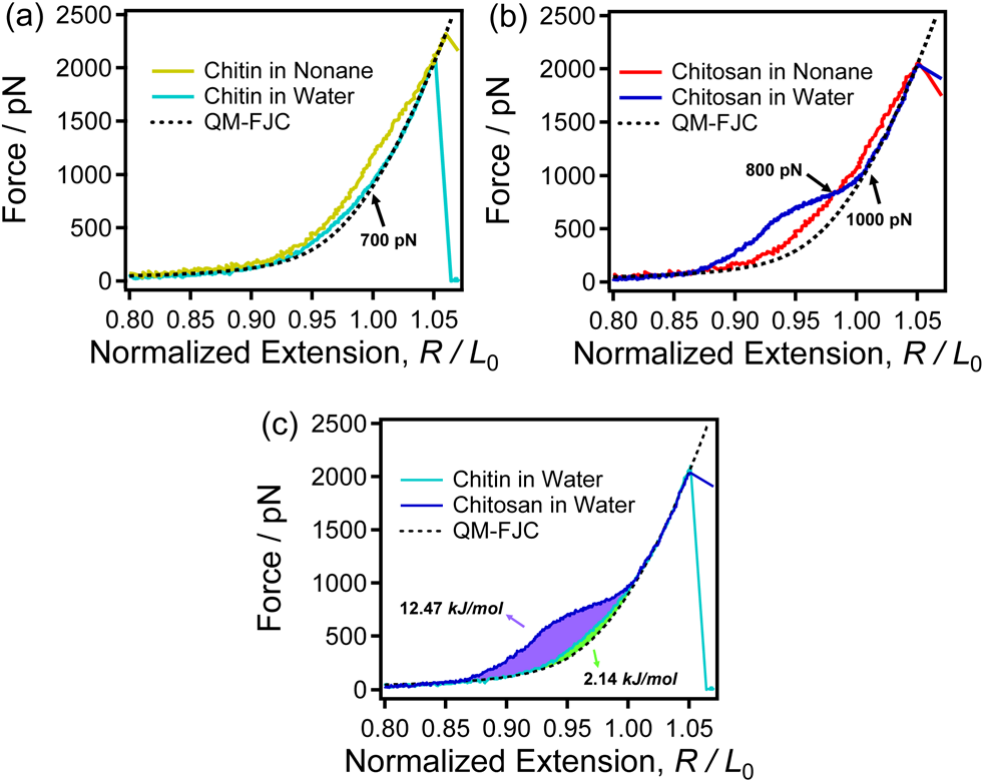
(a) The normalized single-chain F-E curves of chitin obtained in nonane (yellow) and water (light blue) respectively. (b) The normalized single-chain F-E curves of chitosan obtained in nonane (red) and water (blue) respectively. (c) The normalized single-chain F-E curves of chitin (light blue) and chitosan (blue) obtained in water respectively. The black dotted line is the QM-FJC model fitting curve.

Water is the inevitable medium for interaction between biological macromolecules,^65^ whereas both chitin and chitosan are insoluble in water, and mild solubilization methods have been continuously explored. Therefore, studying the role of water in the single molecule properties of these two polysaccharides could provide new ideas for finding mild solubilization methods for them. As shown in Fig. 5 and S3,† the single-chain elasticity of chitin and chitosan differs largely in water.

In Fig. 5a, the curve of chitin in water is between the curve obtained in nonane and theoretical curve. As mentioned before, the strong intrachain H-bonds result that the F-E curve of chitin obtained in nonane (yellow solid line in Fig. 5a) is higher than that of theoretical model fitting curve. Meanwhile, the F-E curve of chitin in water is lower than that obtained in nonane indicating that chitin cannot form strong intrachain H-bonds in water. In addition, the experimental curve of chitin in water is slightly higher than the QM-FJC curve when *F* < 700 pN. The rearrangement of binding water around the polymers (such as amylose and poly(*n*-isopropylacrylamide)) has also been reported to allow the F-E curves of polymer in water to be higher than QM-FJC curve (backbone elasticity).^64^ Thus, it can be speculated that the slight differences between the F-E curve of chitin obtained in water and QM-FJC curve is attributed to the rearrangement of a small amount of binding water or the breakage of a small number of intrachain H-bonds. These results demonstrate that chitin cannot form strong H-bonds (intrachain and with water) in water. In other words, the single-chain mechanics of chitin are insensitive to water.

However, see Fig. 5b, the F-E curve of chitosan obtained in water shows a shoulder-like plateau and forms a crossover with that in nonane at 800 pN. If only intrachain H-bonds are present in water, the intrachain H-bonds structure of chitosan will be weaken by water due to the interaction between water and hydroxyl group/oxygen atom of sugar rings,^51^ then chitosan should present a lower F-E curve in water than that in nonane. Surprisingly, the result was the opposite of what was expected, the F-E curves of chitosan in water are higher than that in nonane when force <800 pN. Thus, the single-chain elasticity of chitosan in water is not only determined by the intrachain H-bonds. Therefore, it can be inferred that there may be other factors contributing more to the single-chain elasticity of chitosan in water. Previous studies have shown that the helical structure and binding water are widely existed in chitosan aqueous solution. The detail of the helical structure of chitosan is dependent on the pH of the environment.^66^ We can speculate that chitosan can form a helical structure in water, which helical structure will be unfolded when stretch chitosan in water (extra energy is needed to stretch chitosan in water). Thus, compared with that in nonane chitosan show a stronger single-chain elasticity in water when <800 pN. Furthermore, beyond 800 pN the F-E curve obtained in water is lower than that in nonane and superposed with the theoretical curve when >1000 pN. Lower than that of the curve obtained in nonane indicating that chitosan cannot form abundant intrachain H-bonds (which can synergistically enhance the single-chain mechanics of chitosan) in water. The superposition demonstrate that chitosan presents the backbone elasticity in this condition, indicating there is no intrachain H-bonds.

The F-E curves of chitin and chitosan in water were compared in Fig. 5c. In high force region, both the F-E curves of chitin and chitosan obtained in water superposed with QM-FJC curve (the backbone elasticity). While, in middle force region (50–1000 pN), the shoulder-like plateau result in the single chain F-E curve of chitosan in water is higher than that of chitin. This is because of the multiple effects of intrachain H-bonds, binding water interactions and the helical structure of chitosan in water, while chitin has no secondary structure and the intrachain H-bonds is weakened in water. The integral calculations show that the energy generated by rearrangement the binding water and unfolding the helical structure of single chitosan is 12.47 kJ mol^−1^ (purple area in Fig. 5c) and the energy consumed to stretch chitin in water is 2.14 kJ mol^−1^ (green area in Fig. 5c). It can be seen that stretching single chitosan chains in water consumes nearly six times more energy than chitosan, which means that the interaction between water and chitosan is stronger than that of chitin. In other words, the affinity for water of these two polysaccharides differs largely. Combine with the previous description, we can draw conclusion that this six times energy is not only used to break the H-bonds but also to induce a conformation change of the helical structure.

In terms of application, the structures and functions of chitosan and chitin are closely related to their affinity for water: the hydrophilicity is necessary for chitosan that serves as the easily modified materials, while the hydrophobicity is very important for chitin that serves as the structural material.^3–5^ The force measurements results demonstrate that the interaction between chitosan and water is stronger than that of chitin. Thus, the single-chain elasticity of chitosan is more sensitive to water than chitin. This may be why chitosan show a better solubility and chemical activity than chitin. Meanwhile, we can conclusion that the affinity for water of chitin is weak than that of chitosan.

The relatively weak affinity for water is a benefit for chitin to maintain the stability of materials, which properties are necessary for chitin serve as structural materials. Chitosan and chitin share the same strength of H-bonds and single-chain elasticities in same organic solvent (nonane or DMSO). However, there is great difference for properties of chitin and chitosan in water. Water plays an important role in regulation the nanomechanics and hydrophobicity of chitin and chitosan at single molecule level, and is deeply involved in the structures and properties of both chitosan and chitin.

## 4 Conclusions

In this paper, the AFM-based SMFS was used to explore the change from chitin to chitosan from a single molecular level. The force measurements study indicate that single-chain elasticity of chitin and chitosan are dependent on the status of H-bonds, which can be affected by the solvent. Chitin and chitosan present the same natural elasticity in nonane, indicating that these two polysaccharides share the same status of H-bonds in a nonpolar organic solvent. The H-bonds of chitin and chitosan can be completely eliminated by dimethyl sulfoxide (DMSO). Thus, chitosan and chitin show the same backbone elasticity in DMSO. However, in water, the energy consumed to stretch single chitosan chain is remarkably larger than that of chitin (6 times). This means that the affinity for water of chitosan is stronger than that of chitin. The single-chain elasticity of chitin is obviously weak than that of chitosan. This means that the nanomechanics of chitin is insensitive to water, which is essential for chitin as a structural material. This study demonstrates that the single molecule mechanics and affinity for water of them can be effectively affected by water through regulation the H-bonds. This study provides a computational approach to calculate the energy of interaction of single polymer chains with surrounding environment and casts new light on the roles of solvent (especially the water) in the properties and functions of polysaccharides.

## Author contributions

Lu Qian: conceptualization, data curation, funding acquisition and writing – original draft. Kai Zhang: investigation and methodology. Xin Guo: formal analysis and validation. Miao Yu: conceptualization, supervision, funding acquisition, writing – review & editing. All authors have read and agreed to the published version of the manuscript.

## Conflicts of interest

There are no conflicts to declare.

## Supplementary Material

RA-013-D2RA07303J-s001
