# Supplementary material for: What happens when chitin becomes chitosan? A single-molecule study
Source: RSC Adv. 2023 Jan 16;13(4):2294–300. doi: 10.1039/d2ra07303j (PMC9841443; doi:10.1039/d2ra07303j)
Supplement: RA-013-D2RA07303J-s001 [file RA-013-D2RA07303J-s001.pdf]

Electronic Supplementary Material (ESI) for RSC Advances.
This journal is © The Royal Society of Chemistry 2023

Supporting Information

What happens when chitin becomes chitosan? A single-molecule study

Lu Qian[2], Kai Zhang[1], Xin Guo[1], Miao Yu[1] *

1 School of Mechanical Engineering, Sichuan University, Chengdu 610065, China;

2 School of Materials Science and Engineering, South China University of Technology, Guangzhou 510641, China;

* Correspondence: miaoyu@scu.edu.cn; Tel.: +86-28-85405307

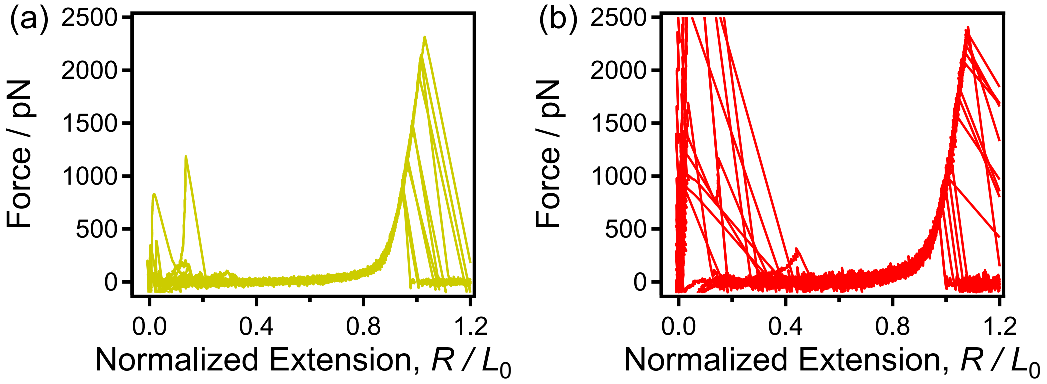

Fig. S1 The typical F-E curves of chitin (a) and chitosan (b) obtain in nonane respectively.

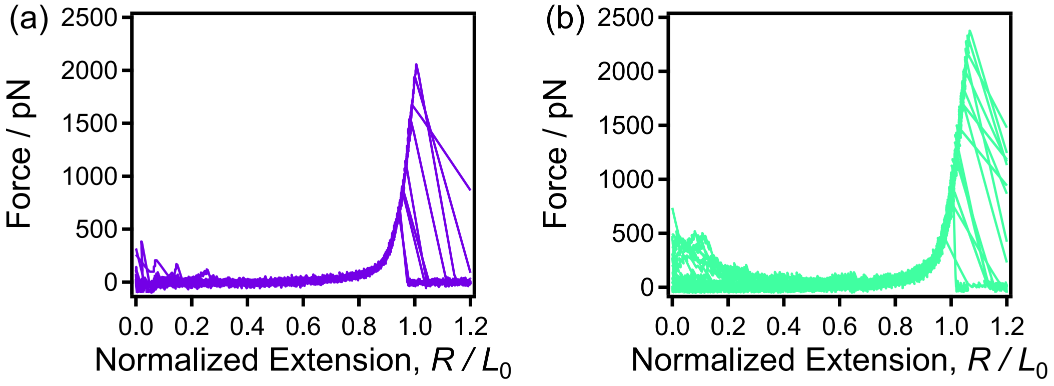

Fig. S2 The typical F-E curves of chitin (a) and chitosan (b) obtain in DMSO respectively.

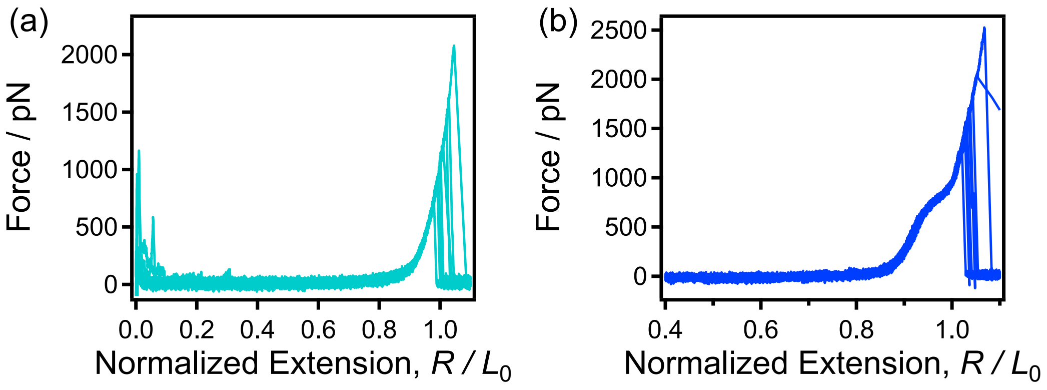

Fig. S3 The normalized single-chain F-E curves of chitin (a) and chitosan (b) obtained in water respectively.
